# Melanogenesis Inhibition by Homoisoflavavone Sappanone A from *Caesalpinia sappan*

**DOI:** 10.3390/ijms130810359

**Published:** 2012-08-20

**Authors:** Te-Sheng Chang, Shih-Yu Chao, Hsiou-Yu Ding

**Affiliations:** 1 Department of Biological Sciences and Technology, National University of Tainan, 33 sec. 2 Su-Lin St., Tainan 700, Taiwan; E-Mail: shawna771126@hotmail.com; 2 Institute of Cosmetics Science, Chia Nan University of Pharmacy and Science, 60 sec. 1 Erh-Jen RD, Jen-Te, Tainan 717, Taiwan

**Keywords:** *Caesalpinia sappan*, inhibition, melanogenesis, sappanone A, tyrosinase

## Abstract

Homoisoflavanone, sappanone A, was isolated from *Caesalpinia sappan* and proven to dose-dependently inhibit both melanogenesis and cellular tyrosinase activity via repressing tyrosinase gene expression in mouse B16 melanoma cells. To our knowledge, sappanone A is the first homoisoflavanone to be discovered with melanogenesis inhibitory activity. Our results give a new impetus to the future search for other homoisoflavanone melanogenesis inhibitors.

## 1. Introduction

The skin color of animals and humans is mainly determined by the content of melanin pigment in the skin, which is produced in dermal melanocytes via a process termed melanogenesis [1]. The process is initiated in melanosomes, the special organelles of melanocytes, with the first and key step of l-tyrosine oxidation to dopaquinone, which is catalyzed by tyrosinase. Although melanin mainly plays a photoprotective role, the accumulation of abnormal amounts of melanin in different parts of the skin, which results in pigmented patches of skin, can also become an esthetic problem. Therefore, several studies have focused on the inhibition of melanogenesis and the prevention of abnormal pigmentation for cosmetic reasons [2–4].

Homoisoflavanones are a small class of naturally occurring oxygen heterocycles [5]. Although these compounds are structurally similar to the more familiar isoflavonoids, their skeleton consists of 16 carbon atoms as opposed to the 15 carbon atoms in the isoflavonoid skeleton. The first homoisoflavanones, eucomin and eucomol, were isolated from *Eucomis bicolor* in 1967. Since then, a variety of homoisoflavanones has been isolated from different plant families across the world. These compounds have been reported to possess anti-inflammatory, antibacterial, antifungal, anti-angiogenic, anticancer, anti-oxidative, antiviral, and anti-phosphodiesterase activities. However, homoisoflavanones have not yet been studied for their anti-melanogenesis activity.

In a preliminary study, we screened crude extracts from 23 Chinese medical herbs to identify their applicability as skin-lightening agents. Among them, the crude extract of *C. sappan* showed strongest inhibitory activity against melanogenesis in mouse B16 melanoma cells. The crude extract of *C. sappan* was evaluated for the antiproliferative activity toward mouse B16 melanoma cells in a previous report [6]. However, results concerning the isolation of active compounds toward antimelanogenesis activity from the plant had not previously been reported. In the present study, the active compound from the extract was isolated and identified by spectrometric methods. In addition, the inhibitory effects of the compound on melanogenesis were studied in B16 cells.

## 2. Results and Discussion

**Figure 1:**
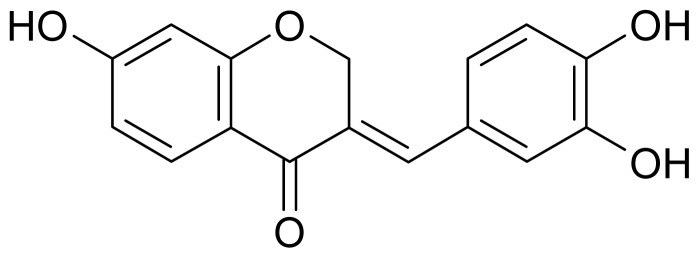
Chemical structure of sappanone A.

In our continued search for new natural melanogenesis inhibitors, we found the methanol extract of *C. sappan* showed strong inhibitory activity against melanogenesis in B16 cells. Following bioassay-guided purification of the methanol extract by methanol extraction, *n*-hexane, ethyl acetate, *n*-BuOH, and water partitioning, and repeated silica gel column-chromatography methods, one active compound was ultimately isolated. The chemical structure of the isolated compound was determined by mass spectra, ^1^H-NMR, and ^13^C-NMR analysis, and was identified as sappanone A (Figure 1) by comparing these data with those in the literature [7]. The compound has also been isolated from the heartwood of *C. japonica* [8] and *C. pulcherrima* [9]. In the previous studies, sappanone A was proven to possess anti-oxidative, antibacterial, and antifungal activities [9,10]. However, the anti-melanogenesis activity of sappanone A has not yet been evaluated.

**Figure 2:**
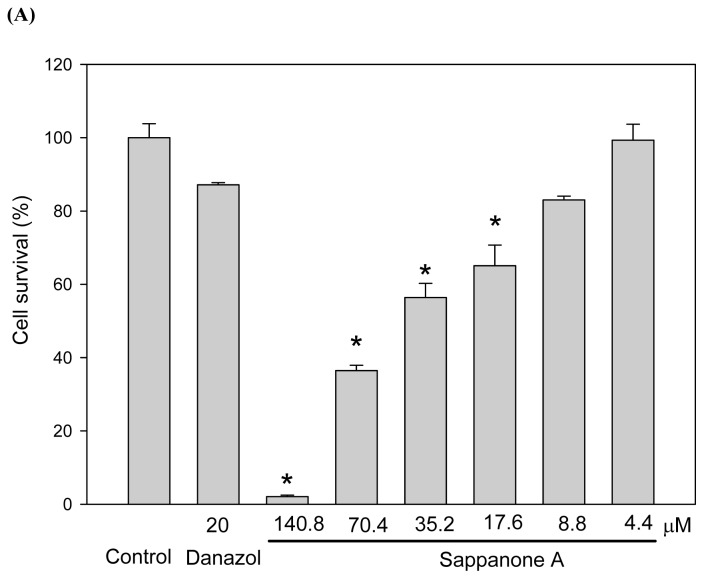

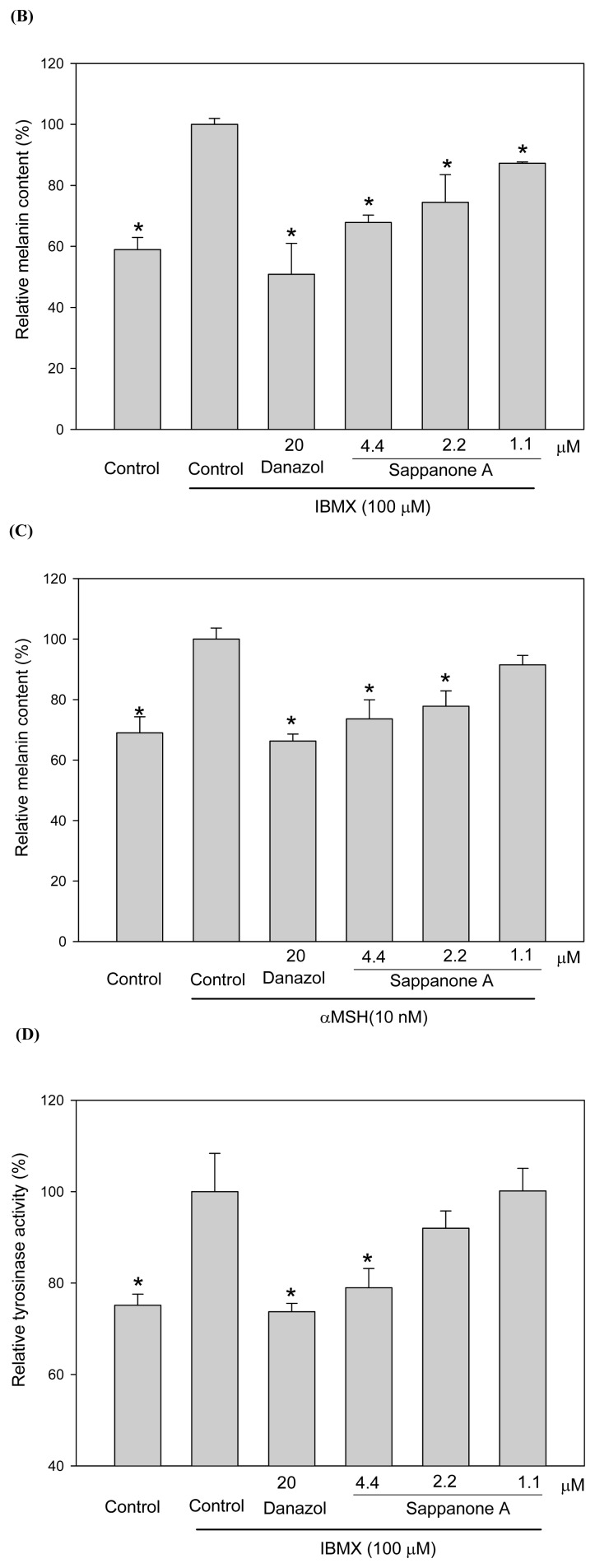
Effects of sappanone A on cell survival (**A**), melanin content (**B**, **C**), and cellular tyrosinase activity (**D**) in mouse B16 melanoma cells. The cells were seeded in 24-well plates for 1 day and then treated with various dosages of sappanone A for 2 days. Cell viability was then examined by a MTT assay (**A**), and both melanin content (**B**, **C**) and cellular tyrosinase activity (**D**) of the cells were determined using spectrometry, according to the work by Lin *et al.* [3]. The average data (*n* = 3) is presented with an error bar of S.D. A value of *p* < 0.01 (*) from a Student’s *t*-test analysis by comparing the data with that of the control (**A**), the IBMX-stimulated control (**B**, **D**), or the αMSH-stimulated control (**C**) was considered to be statistically significant.

We used mouse B16 melanoma cells to study melanogenesis inhibition by sappanone A. Figure 2A shows the cytotoxicity of the compound toward the cells. We found sappanone A at concentrations of 8.8 μM had no significant cytotoxic effects on the cells. In order to evaluate the melanogenesis inhibition precisely, we used 4.4 μM of sappanone A as the maximal concentration for the depigmenting assay to avoid the interference of cytotoxicity. At the beginning of the study, we used both α melanocyte stimulating hormone (αMSH) and 3-isobutyl-1-methylxanthin (IBMX), an agent that stimulates intracellular cAMP levels, to stimulate melanogenesis in B16 cells. As shown in Figure 2B,C, the melanin content of the B16 cells increased considerably after stimulation with both αMSH and IBMX. As low as 1.1 μM of sappanone A treatment resulted in significant prevention of the increase in melanin content induced by IBMX in the B16 cells. The inhibition of melanogenesis by sappanone A was also dose-dependent, where the inhibition of the treatment by 4.4 μM of sappanone A was comparable to that of the treatment by 20 μM of danazol, which has been proven to be a potent melanogenesis inhibitor [11]. In addition, sappanone A treatment also resulted in a dose-dependent decrease in cellular tyrosinase activity, the key enzyme involved in melanogenesis (Figure 2D). The levels of the residual quantities of melanin and tyrosinase activity in the cells treated with 4.4 μM of sappanone A are 67.8% ± 2.4% (Figure 2B) and 78.9% ± 4.2% (Figure 2D), respectively, compared to those in the IBMX-treated control cells. Hence, the inhibitory levels of sappanone A on melanogenesis and tyrosinase activity are 32.2% and 21.1%, respectively. It is reasonable that melanogenesis is inhibited with the level of 32.2% while cellular tyrosinase activity is reduced with the level of 21.2%. The reduction in cellular tyrosinase activity by sappanone A was thought to be attributable to either the direct inhibition of tyrosinase activity or the repression of tyrosinase gene expression. However, the former possibility was excluded by direct enzyme activity assay, where no enzyme activity inhibition was observed within the tested concentration range of sappanone A (data not shown).

**Figure 3:**
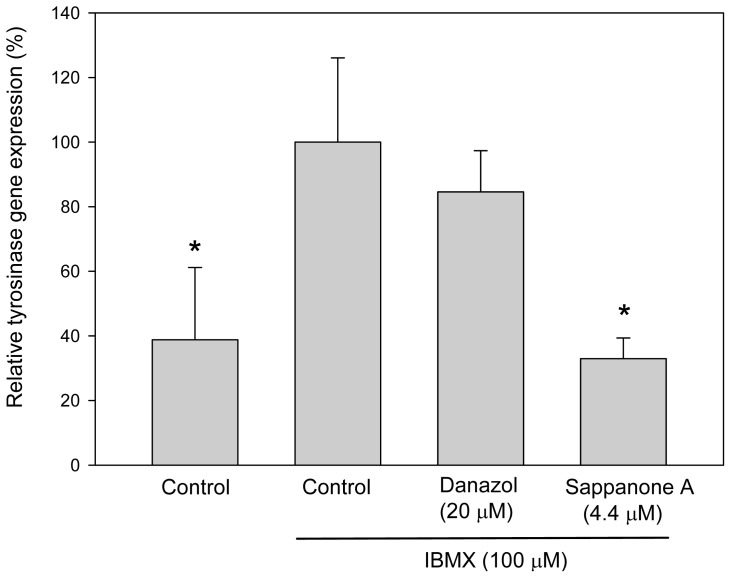
Effects of sappanone A on tyrosinase gene expression in B16 cells. The cells were cultivated for 1 day and then stimulated with 100 μM of IBMX for 2 days in the presence to the indicated drugs. The mRNA levels of the cells were determined using qRT-PCR methods, as described in the publication by Lin *et al.* [3]. The average data (*n* = 3) is presented with an error bar of S.D. A value of *p* < 0.05 (*) from a Student’s *t*-test analysis by comparing the data with that of the IBMX-stimulated control was considered to be statistically significant.

From the above resutls, sappanone A appeared to inhibit melanogenesis in B16 cells by inhibiting tyrosinase gene expression. To investigate whether sappanone A inhibits tyrosinase gene expression, we evaluated gene expression by assessing the levels of tyrosinase mRNA via real-time reverse-transcription polymerase chain reaction (qRT-PCR) assays. As we expected, sappanone A-treated cells contained a lower levels of tyrosinase mRNA compared with those in the IBMX-stimulated control cells (Figure 3). In contrast, danazol did not downregulate the levels of tyrosinase mRNA in the cells due to the post-transcriptional regulation effects on tyrosinase gene expression as proven before [11]. From the results above, we concluded that sappanone A inhibits both melanogenesis and cellular tyrosinase activity in mouse B16 melanoma cells via repressing tyrosinase gene expression.

The biosynthetic pathway of melanogenesis has been elucidated clearly [1]. Melanogenesis is initiated with the first step of tyrosine oxidation to dopaquinone catalyzed by the key enzyme, tyrosinase. This first step is the rate-limiting step in melanin synthesis because the remainder of the reaction sequence can proceed spontaneously at a physiological pH value. Although three enzymes (tyrosinase, tyrosinase related protein 1 and 2) are involved in melanogenesis pathway, only tyrosinase is absolutely necessary for melanogenesis due to the key role in melanogenesis. Hence, almost every melanogenesis inhibitor identified so far achieves the inhibitory activity via reducing cellular tyrosinase activity. In the present manuscript, we evaluated the melanogenesis inhibitory effects of sappanone A and investigated the inhibitory effects on both cellular tyrosinase activity and tyrosinase gene expression in advance. We found that sappanone A dose-dependently inhibits both melanogenesis and cellular tyrosinase activity via repressing tyrosinase gene expression in mouse B16 melanoma cells. We focused on the key enzyme tyrosinase in the present study. However, the possibility cannot be ruled out that sappanone A could also affect other gene expressions in B16 cells and the interesting work should be studied in the future.

In the past few years, dozens of melanogenesis inhibitors have been discovered from natural sources. Among them, many potent inhibitors belong to the isoflavonoids [4,12–15]. From the perspective of structure and activity relationships (SARs), some homoisoflavanones, which have similar structures to those of isoflavonoids, are reasonably thought to possess melanogenesis inhibitory activity. However, in spite of multifunctional bioactivities of homoisoflavanones, including anti-inflammatory, antibacterial, antifungal, anti-angiogenic, anticancer, anti-oxidative, antiviral, and anti-phosphodiesterase activities, have been identified, homoisoflavanones have not yet been studied for their anti-melanogenesis activity [5]. In the present study, our results confirmed that the homoisoflavanone, sappanone A, strongly inhibited both melanogenesis and cellular tyrosinase activity in cultured B16 melanoma cells via repressing tyrosinase gene expression. To the best of our knowledge, sappanone A is the first homoisoflavanone to be discovered with melanogenesis inhibitory activity. Because sappanone A is the first homoisoflavanone compound to be identified as a melanogenesis inhibitor, we would surmise that other homoisoflavanones also act in a similar manner, but they have not yet been evaluated. However, due to lack of other homoisoflavanones available in our laboratory, we cannot compare the inhibitory effects of sappanone A with those of other homoisoflavanones. Therefore, it would seem worthwhile to search new melanogenesis inhibitors from other homoisoflavanones-containing plants.

## 3. Experimental Section

### 3.1. Materials

3-(4,5-Dimethylthiazol-2-yl)-2,5-diphenyltetrazolium bromide (MTT), Triton X-100, phenylmethylsulfonyl fluoride (PMSF), l-DOPA (l-3,4-dihydroxyphenylalanine), dimethyl sulfoxide (DMSO), trypsin/EDTA, αMSH, and IBMX were purchased from Sigma (St. Louis, MO). Danazol was bought from Tokyo Chemical Industry Co. (Tokyo, Japan). Protease inhibitor cocktail was obtained from Abcam (Cambridge, MA). All other chemicals were obtained from Tokyo Chemical Industry (Tokyo) and were of analytic reagent grade.

### 3.2. Isolation of Sappanone A from *C. sappan*

The dry powder of *C. sappan* heartwood (33.0 kg) was extracted with 95% ethanol at room temperature. After removal of the solvent by evaporation, the residue (3.45 kg) was partitioned with water and ethyl acetate (1:2). The ethyl acetate layer was removed by evaporation and the residue was then suspended in methanol-water (9.5:0.5) and partitioned with *n*-hexane (1:1). The methanol layer was subjected to silica gel column chromatography and eluted with *n*-hexane-ethyl acetate (7.5:2.5, 1:1, 2.5:7.5), ethyl acetate, ethyl acetate-methanol (1:1), and methanol, successively. Each fraction collected from the column was monitored by thin-layer chromatography and the similar fractions were combined to produce 8 fractions. The fraction 3 was further purified by LH-20 Sephadex and eluted with methanol to isolate sappanone A (137.7 mg). The structure was confirmed by NMR and mass spectra analysis.

Sappanone A: yellow powder; m.p. 221–222 °C; ESI/MS *m/z*: 283 [M-H]^+; 1^H-NMR (acetone-d_6_, 500 MHz) *δ*: 5.39 (2H, d, *J* =2.0 Hz, H-2), 6.37 (1H, d, *J* =2.0 Hz, H-8), 6.59 (1H, dd, *J* =9.0, 2.0 Hz, H-6), 6.82 (1H, dd, *J* =8.0, 2.0 Hz, H-6′), 6.92 (1H, d, *J* =2.0 Hz, H-2′), 6.93 (1H, d, *J* =8.0 Hz, H-5′), 7.62 (1H, br s, H-9), 7.81(1H, d, *J* = 9.0 Hz, H-5); ^13^C-NMR (acetone-d_6_,125 MHz) *δ*:180.8 (C-4, C=O), 165.3 (C-7), 163.9 (C-8a), 148.0 (C-3′), 146.2 (C-4′), 137.0 (C-9), 130.4 (C-5), 129.5 (C-1′), 127.5 (C-3), 124.2 (C-6′), 118.1 (C-5′), 116.4 (C-2′), 116.0 (C-4a), 111.8 (C-6), 103.5 (C-8), 68.8 (C-2). These data were compared with literature values [7].

### 3.3. Cell Cultures and Drug Treatments

Mouse B16 melanoma cells (4A5) were obtained from the Bioresources Collection and Research Center (BCRC, Food Industry Research and Development Institute, Hsinchu). The cells were cultured in Dulbecco’s modified Eagle’s medium (DMEM) supplemented with 10% (*v*/*v*) fetal bovine serum at 37 °C in a humidified, CO_2_-controlled (5%) incubator. The cells were seeded at an appropriate cell density in a 24-well or a 6-well plate. After 1 day of incubation, the cells were treated with various concentrations of drugs in the absence or presence of a stimulation agent (100 μM of IBMX) for another 2 days. Thereafter, the cells were harvested and used for various assays.

### 3.4. Measurements of Cell Viability

MTT assays were performed to determine cell viability. After the cells were incubated with the drugs for 48 h, the culture medium was removed and replaced with 1 mg/mL MTT solution dissolved in phosphate-buffered saline (PBS) for a further 2 h incubation. The MTT solution was then removed, DMSO was added, and the absorbance of the dissolved formazan crystals was determined at 570 nm.

### 3.5. Determination of Melanin Content

At the end of cell cultivation, the cells were harvested and washed twice with PBS. The pelleted cells were lysed in repeated frozen in lysis buffer containing 20 mM sodium phosphate (pH 6.8) and 1% Triton X-100. After centrifugation at 15,000× *g* for 15 min, the melanin pellets were dissolved in 1 N NaOH containing 20% DMSO for 1 h at 95 °C. The melanin content was measured by the absorbance at 490 nm.

### 3.6. Measurements of Cellular Tyrosinase Activity

Tyrosinase activity in B16 cells was examined by measuring the rate of oxidation of l-DOPA. The drug-treated cells were washed with ice-cold PBS and lysed with 20 mM phosphate buffer (pH 6.8) containing 1% Triton X-100 and 1 mM PMSF. Detergent was used to release the membrane-bound tyrosinase from the melanosomes. Cells were then disrupted by freezing and thawing. The lysates were centrifuged at 15,000× *g* for 15 min. Tyrosinase activity was then determined as follows: 1 mL of the reaction mixture contained 50 mM of phosphate buffer (pH 6.8), 2.5 mM of l-DOPA, and 500 μg of the supernatant protein. After a 15 min reaction at 37 °C, dopachrome formation was monitored by measuring absorbance at 475 nm.

### 3.7. Quantitative Real-Time Reverse Transcription Polymerase Chain Reaction (Real Time qRT-PCR)

Real time qRT-PCR were performed on the ABI 7500 Real Time PCR system (Applied Biosystems, Foster, CA) using Fast SYBR^®^ Green Master Mix (Applied Biosystems). Total RNA was extracted using an RNeasy^®^ mini Kit (Qiagen, Valencia, CA) according to the manufacturer’s instructions. The quality of the total RNA sample was evaluated by determining the OD_260_/OD_280_ ratio. To prepare a cDNA pool from each RNA sample, total RNA (2 μg) was reverse transcribed at 42 °C for 90 min in the presence of oligo(dT) primers (MD Bio., Taipei) and reverse transcriptase (Roche Molecular Biochemicals, Mannheim). The oligonucleotides primers for mouse tyrosinase (forward, 5′-GGCCAGCTTTCAGGCAGAGGT-3′; reverse, 5′-TGGTGCTTCATGGGCAAAATC-3′) and mouse glyceraldehyde-3-phosphate dehydrogenase (GAPDH) as an internal control (forward, 5′-ACCACAGTCCATGCCATCAC-3′; reverse, 5′-TCCACCACCCTGTTGCTGTA-3′) were used. After the initial incubation of 2 min at 50 °C, the cDNA was denatured at 95 °C for 10 min followed by 40 cycles of PCR (95 °C, 15 s, 60 °C, 60 s). All results were obtained from at least three independent experiments. The mRNA level of tyrosinase was normalized using GAPDH as an internal control. The relative tyrosinase mRNA content was calculated by dividing the normalized data by that from the IBMX-stimulated control reaction.

### 3.8. Statistical Analysis

All of the data in the present study were obtained as averages of experiments that were performed at least in triplicate and are expressed as means ± SD Statistical analysis was performed by the Student’s *t* test. A value of *p* < 0.05 or *p* < 0.01 was considered to be statistically significant.

## 4. Conclusions

In conclusion, our results not only demonstrate that sappanone A is the first homoisoflavanone to be discovered with melanogenesis inhibitory activity, but also give a new impetus to the future search for other homoisoflavanone melanogenesis inhibitors.
